# A discriminant analysis of plasma metabolomics for the assessment of metabolic responsiveness to red raspberry consumption

**DOI:** 10.3389/fnut.2023.1104685

**Published:** 2023-03-23

**Authors:** Valentin Barbe, Juan de Toro-Martín, Rodrigo San-Cristobal, Véronique Garneau, Geneviève Pilon, Patrick Couture, Denis Roy, Charles Couillard, André Marette, Marie-Claude Vohl

**Affiliations:** ^1^Centre Nutrition, santé et société (NUTRISS), Université Laval, Québec City, QC, Canada; ^2^Institut sur la nutrition et les aliments fonctionnels (INAF), Université Laval, Québec City, QC, Canada; ^3^School of Nutrition, Université Laval, Québec City, QC, Canada; ^4^Québec Heart and Lung Institute (IUCPQ) Research Center, Québec City, QC, Canada; ^5^Endocrinology and Nephrology Unit, CHU de Quebec Research Center, Québec City, QC, Canada

**Keywords:** raspberry, clustering, machine learning, metabolic health, metabolomics, precision nutrition

## Abstract

**Background:**

Many studies show that the intake of raspberries is beneficial to immune-metabolic health, but the responses of individuals are heterogeneous and not fully understood.

**Methods:**

In a two-arm parallel-group, randomized, controlled trial, immune-metabolic outcomes and plasma metabolite levels were analyzed before and after an 8-week red raspberry consumption. Based on partial least squares discriminant analysis (PLS-DA) on plasma xenobiotic levels, adherence to the intervention was first evaluated. A second PLS-DA followed by hierarchical clustering was used to classify individuals into response subgroups. Clinical immune and metabolic outcomes, including insulin resistance (HOMA-IR) and sensitivity (Matsuda, QUICKI) indices, during the intervention were assessed and compared between response subgroups.

**Results:**

Two subgroups of participants, type 1 responders (*n* = 17) and type 2 responders (*n* = 5), were identified based on plasma metabolite levels measured during the intervention. Type 1 responders showed neutral to negative effects on immune-metabolic clinical parameters after raspberry consumption, and type 2 responders showed positive effects on the same parameters. Changes in waist circumference, waist-to-hip ratio, fasting plasma apolipoprotein B, C-reactive protein and insulin levels as well as Matsuda, HOMA-IR and QUICKI were significantly different between the two response subgroups. A deleterious effect of two carotenoid metabolites was also observed in type 1 responders but these variables were significantly associated with beneficial changes in the QUICKI index and in fasting insulin levels in type 2 responders. Increased 3-ureidopropionate levels were associated with a decrease in the Matsuda index in type 2 responders, suggesting that this metabolite is associated with a decrease in insulin sensitivity for those subjects, whereas the opposite was observed for type 1 responders.

**Conclusion:**

The beneficial effects associated with red raspberry consumption are subject to inter-individual variability. Metabolomics-based clustering appears to be an effective way to assess adherence to a nutritional intervention and to classify individuals according to their immune-metabolic responsiveness to the intervention. This approach may be replicated in future studies to provide a better understanding of how interindividual variability impacts the effects of nutritional interventions on immune-metabolic health.

## 1. Introduction

It has been shown that obesity and metabolic syndrome increase type 2 diabetes (T2D) incidence and cardiovascular disease morbidity and mortality rates (1). With both environmental and biological factors affecting the risk of an individual to develop T2D (2), the beneficial effects of plant-based diets on metabolic health have been previously highlighted (3). The consumption of fruits, and in particular berries, has been associated with beneficial health effects, especially in the prevention of metabolic disturbances (4). Berries have been consumed since the roman empire and were used to treat diseases in medieval Europe (5).

These fruits are natural source of dietary fiber and many other nutrients and phytochemicals with beneficial health properties. Berries are rich in numerous polyphenols, classified as flavonoids and non-flavonoids, which have favorable effects on obesity, hypertension, dyslipidemia and hyperglycemia, at least in part through their potential antioxidant and anti-inflammatory properties (6). In particular, the polyphenolic content and antioxidant activity of raspberries are ranked among the highest of commonly consumed fruits (7). Moreover, studies have shown that most consumed berries such as raspberries improve postprandial hyperglycemia and hyperinsulinemia in individuals with overweight or obesity, as well as with metabolic syndrome, suggesting that these fruits may have a beneficial impact on type 2 diabetes prevention and management (8, 9).

The inclusion of metabolomics-based plasma metabolic profiling has allowed the identification of nutritional markers related with intervention adherence and health response (10). The human plasma metabolome contains hundreds of circulating metabolites reflecting the physiology, genetics, environmental exposures and dietary habits of individuals (11). These metabolites include mainly xenobiotics, lipids, amino acids, vitamins and cofactors, and nucleotides. In this regard, while most of past research has demonstrated the beneficial effects of raspberry consumption on health parameters, few studies have focused on analyzing the metabolic response to raspberries through metabolomics. The main goals of the present study were to identify different types of metabolic responses to an 8-week raspberry consumption based on the plasma metabolomics signature of participants and to develop a framework for assessing adherence to a nutritional intervention’s guidelines.

## 2. Materials and methods

### 2.1. Study design and participants

The study design consisted of a two-arm parallel-group, randomized, controlled trial of the effects of raspberry consumption on the metabolic parameters and plasma metabolome in subjects with metabolic disturbances. The trial, registered as NCT03620617 at clinicaltrials.gov, took place from 2018 to 2019 at the Institute of Nutrition and Functional Foods (INAF) at Université Laval. The written consent was obtained for all participants after the study was approved by the Université Laval Ethics Committee (CER-Université Laval 2017-218). Study participants were men or pre-menopausal women aged between 18 to 60 years old, with a body mass index (BMI) ranging from 25 to 40 kg/m^2^ or a waist circumference greater or equal to 94 cm for men and 80 cm for women. After eligibility was confirmed and a 2-week run-in-period, subjects were randomly instructed to consume 280 g of frozen red raspberries per day (*n* = 24) or to maintain their usual diet (*n* = 25) for 8 weeks. Nutritional and clinical data of participants were collected from food frequency questionnaires (FFQ), medical questionnaires and physical examinations (12). Blood samples were taken before (week 0) and after the 8 weeks (week 8) of raspberry consumption. We have summarized the nutritional composition of raspberries in Supplementary Table 1. All data is representative of two cups of raspberries (4 portions), which participants consumed daily for 8 weeks. Further details on this clinical study are available in (12). For the present study, data from the 24 subjects of the group consuming raspberries were used. In addition to the clinical variables available in the clinical study (12), the quantitative insulin sensitivity check index (QUICKI) was computed for all participants using 1/[log_10_(fasting insulin) + log_10_(fasting glucose)] (13). Matsuda index is used to evaluate insulin sensitivity from the data obtained by an oral glucose tolerance test (14). Homeostatic model assessment for insulin resistance (HOMA-IR) is calculated from fasting glucose and fasting insulin levels and is an index widely used to assess insulin resistance in individuals (15).

### 2.2. Plasma metabolome profiling

Targeted metabolomics using ultra-performance liquid chromatography–tandem mass spectrometry on the Metabolon DiscoveryHD4^®^ platform (Morrisville, NC, United States) were performed on fasted plasma samples of the 24 participants of the raspberry group collected before (week 0) and after (week 8) the raspberry consumption (16). The dataset of metabolites consisted of a total of 1,132 biochemicals which included lipids, amino acids, xenobiotics, cofactors and vitamins, nucleotides, carbohydrates and peptides. Data were normalized by dividing the raw values in the experimental batch by the median of those samples in each instrument batch, giving each batch and thus the metabolite a median of one. After batch normalization, data were further imputed by replacing missing values for a given metabolite with its observed minimum. This was done to avoid inflating the false negative rate and weaken the statistical power of the analyses. Normalized and imputed data were then transformed using natural log and filtered based on inter-individual variance. Metabolites presenting no variance (*n* = 14) or low variance (< 0.1; *n* = 272) were excluded from further analyses. Data were further filtered to remove unknown compounds (216 unnamed biochemicals).

### 2.3. Xenobiotics and adherence to the nutritional intervention

Metabolites in the Metabolon dataset classified as “xenobiotics” were used herein as metabolites reflecting the adherence of participants to the nutritional intervention. Partial least squares discriminant analysis (PLS-DA) is a supervised classification algorithm reducing the dimensionality of the data to analyze the covariance between categorical dependent variables and a very large number of independent variables. A first sparse PLS-DA (sPLS-DA) was done to confirm adherence to the nutritional intervention by discriminating trial visits, before and after raspberry consumption, using only xenobiotics (*n* = 120; Supplementary Figure 1). To identify participants with a low adherence to the protocol, we performed a second step based on the prediction of the intervention timepoints. For training and testing data, the initial dataset was split in two equal sets containing the same number of samples and equal proportion of men and women. A trained sPLS-DA model was used to predict the intervention timepoint (pre-or post-raspberry consumption) of plasma samples. This was first done while including participants whose adherence was considered as low, and then after excluding them. It served the purpose of confirming whether their exclusion from the dataset was justified by examining prediction performance statistics of the model and the model’s error rate. PLS-DAs, sPLS-DAs and classification performance evaluation of the models were computed using the mixOmics R package (v6.20.0) (17).

### 2.4. Clustering

Metabolites for clustering analysis were filtered by removing xenobiotics (*n* = 120) and partially characterized molecules (*n* = 24). A total of 486 out of the 1,132 initial metabolites reflecting the participants’ endogenous response to the intervention were used for this analysis, as shown in Supplementary Figure 1. A PLS-DA was then used in combination with a hierarchical clustering analysis (HCA) to identify clusters of participants with distinct metabolic response to raspberry consumption. The PLS-DA model was instructed to discriminate plasma samples belonging to pre-versus post-raspberry consumption timepoints. In order to identify response subgroups, the two main components resulting from the PLS-DA were used as input data for the HCA, with Euclidean distance and Ward linkage as the main parameters of the model. This was done using pvclust R package (v2.2.0) (18), which calculates approximately unbiased value of ps for all clusters by using multiscale bootstrap resampling (*n* = 1,000 replications). An unbiased value of p of 95% or above was considered to robustly support the identified clusters. Finally, a sPLS-DA was performed considering the newly identified clusters of participants. The sPLS-DA was done to strengthen the classification and identify the most discriminating metabolites in each subgroup. The optimal number of metabolites to use was determined during the tuning process, which was run using 10-fold cross validation and 20 repeats. A multilevel approach was used to correctly assess the structure of the data, which includes two timepoints per participant (before and after the intervention). The stability of selected metabolites within each component was computed as the proportion of folds where the loading was used to assess a given component during cross validation. The model’s performance was then evaluated using the built-in tools to estimate the classification error rate.

### 2.5. Statistical analyses

A two-tailed unpaired t-test was first used to compare baseline characteristics between subgroups at week 0. We then explored the metabolic homogeneity of participants within each subgroup and the heterogeneity between subgroups, and assessed the physiological relevance of the metabolomics-based raspberry responsiveness classification. To do that, a linear mixed model using the nlme (v3.1.157) (19) and emmeans (v1.7.5) (20) R packages was used to compare the changes in metabolic parameters in response to raspberry consumption between subgroups. This model was used to test the effects of group, timepoint and their interaction considering the effects of age and sex. A second linear mixed model was used to test the association between plasma metabolite levels and clinical data at weeks 0 and 8. The 10 most discriminant metabolites of components 1 and 2 and the clinical variables for which the differences between subgroups were significant were used in this model. When significant interactions at *p* ≤ 0.05 between metabolites and clinical variables were observed, a contrast analysis was performed to test for differences between groups.

## 3. Results

### 3.1. Adherence confirmation by xenobiotic-based PLS-DA

Xenobiotics found in the metabolome of participants were useful for identifying participants with a low adherence to the study protocol. Based on the levels of certain xenobiotics, the sPLS-DA revealed two potential non-adherent participants (Supplementary Figure 2A). The most discriminant xenobiotics were methyl glucopyranoside, 4-acetylphenyl sulfate and dihydrocaffeate sulfate (Supplementary Figure 2B). To confirm the outlier status of these two participants, we predicted the intervention timepoint. When removing these two participants, we achieved a prediction accuracy of 100%. By including these two participants, the accuracy decreased to 75%, and both subjects were systematically misclassified. For this reason, these two participants were removed from all further analyses, leaving a total of 22 study participants.

### 3.2. Hierarchical clustering of response subgroups to the raspberry consumption

**Figure 1:**
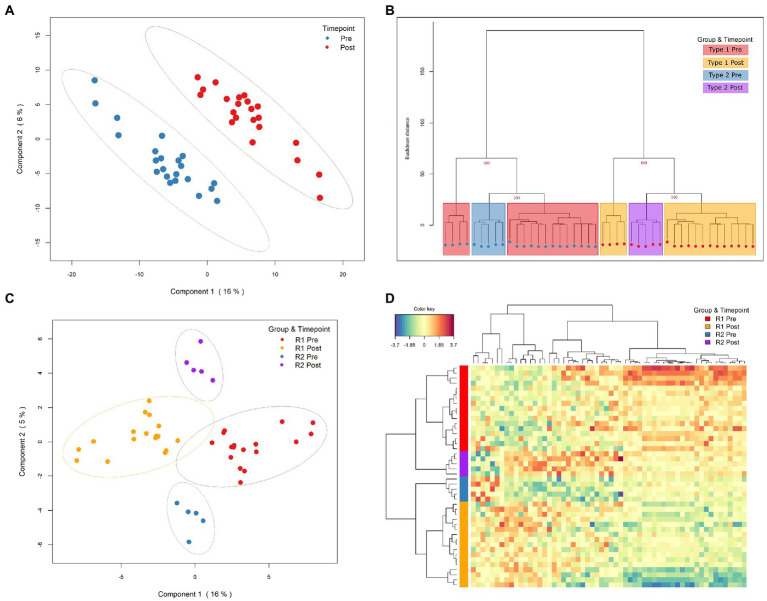
Main steps of the metabolomic-based clustering procedure. Panel **(A)** shows participants spanned by the two main components derived from partial least squares discriminant analysis (PLS-DA) grouped by timepoint (pre- and post-intervention, respectively in blue and red). Each ellipse represents the 95% confidence interval for each timepoint group. Panel **(B)** shows the four clusters of participants identified from hierarchical clustering analysis (HCA). Red and orange squares regroup type 1 responders at pre- and post-intervention timepoints. Blue and purple squares represent type 2 responders at pre-and post-intervention timepoints. Numbers in red represent the approximately unbiased *p*-values of each cluster. Panel **(C)** shows participants spanned by the two main components derived from sparse PLS-DA portraying the two distinct response subgroups identified from HCA. R1 pre-and R1 post-intervention subgroups are colored in red and orange, respectively. R2 pre- and R2 post-intervention subgroups are colored in blue and purple, respectively. **(D)** Heatmap illustrating the classification of participants based on the most discriminating metabolites derived from sparse partial least squares discriminant analysis (sPLSDA). The left dendrogram branches in four major nodes, representing the clustering of participants. The upper dendrogram branches in two major nodes, representing the first component with its 30 metabolites on the right, and the second component with its 30 metabolites on the left. The intensity of red color indicates an increase in metabolite levels between pre-and post-intervention timepoints.

The first two components of the PLS-DA aimed at discriminating between intervention timepoints with endogenous metabolites accounting for, respectively, 16 and 6% of the variance (Figure 1A). The HCA on the two latent variables derived from the PLS-DA revealed subgroups of matched participants with homogenous and well-discriminated metabolomic profiles at pre- and post-intervention visits, with approximately unbiased *p*-values greater than 95% (Figure 1B). Two clusters of participants were discriminated based on component 1 and were considered as type 1 responders (*n* = 17). Another two subgroups of participants were discriminated based on component 2 (*n* = 5) and were considered as type 2 responders (Figure 1B).

A multilevel sPLS-DA model was then built using these two subgroups as an input to determine which metabolites were the most discriminant and to discover the optimal number of metabolites to use in each component (Figure 1C). From this sPLS-DA, component 1 accounted for 16% of variance and was composed of 30 metabolites whereas component 2 accounted for 5% of variance and also included 30 metabolites. A heatmap illustrating these results is shown in Figure 1D. Performance evaluation of the model showed an average classification error rate of around 26% (Supplementary Figure 3A). The stability of the selected metabolites is shown in Supplementary Figure 3B. We observed a high stability for most discriminant metabolites in components 1 and 2. The top 10 metabolites in component 1 all have a stability of 0.90 or higher, while component 2 top 10 metabolites ranged from 0.98 to 0.74. This shows that metabolites in both components are highly discriminative.

### 3.3. Physiological relevance of clustering

We observed no significant differences between type 1 and type 2 responders for age, sex, body weight, BMI and all other clinical parameters at week 0 (Supplementary Table 2).

**Figure 2:**
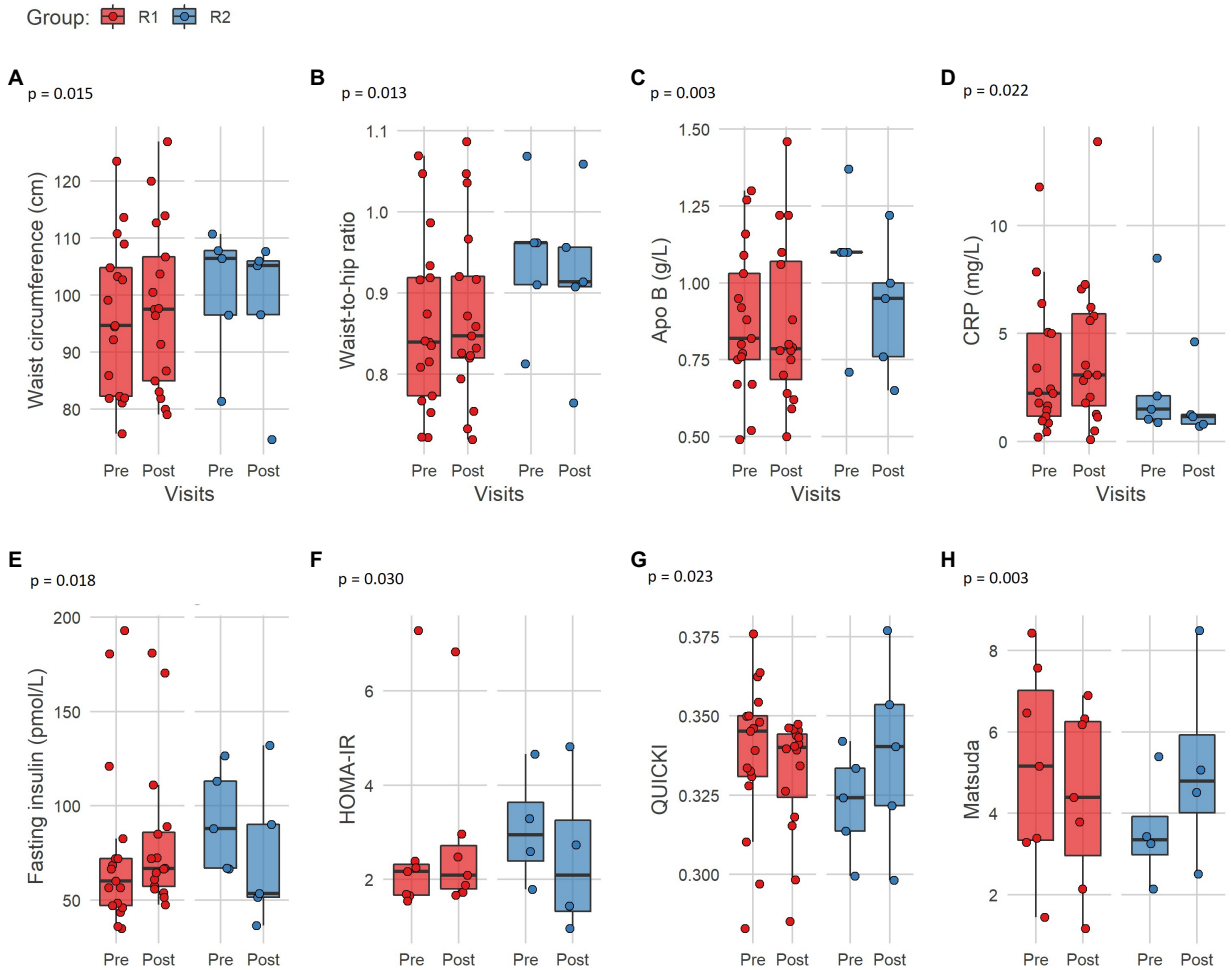
Metabolic differences between pre- and post-intervention by response subgroup. Panels **(A) – (H)** show all significant differences in metabolic parameters between pre- and post-intervention timepoints for type 1 responders (red) and type 2 responders (blue) derived from a linear mixed model. *p* Values shown above each panel represent the *p* for group × visit interaction. Differences accounted for age and sex and resulted from the interaction between the effects of group and timepoint. Each point represents a participant. The mean is represented by the horizontal line, and the standard error is represented by the vertical lines. QUICKI, Matsuda, HOMA-IR and waist-to-hip ratio have no unit.

Changes in all clinical parameters between weeks 0 and 8 for type 1 and type 2 responders are shown in Table 1 and all the significant visit-by-group interactions are shown in Figure 2. As compared to type 1 responders, type 2 responders showed a significant decrease in waist circumference (p for group x visit interaction, p_i_ = 0.02; Figure 2A), waist-to-hip ratio (p_i_ = 0.01; Figure 2B), plasma apolipoprotein B (ApoB; p_i_ = 0.003; Figure 2C), C-reactive protein (CRP; p_i_ = 0.02; Figure 2D), fasting insulin levels (p_i_ = 0.02; Figure 2E), and HOMA-IR (p_i_ = 0.03; Figure 2F) and a significant increase in the QUICKI index (p_i_ = 0.02; Figure 2G) and Matsuda (p_i_ = 0.003; Figure 2H).For most clinical parameters, we observed the opposite effect in type 1 responders, with fasting insulin, waist-to-hip ratio and plasma CRP levels being higher than baseline after the intervention, while QUICKI and Matsuda indices were lower. Waist circumference, HOMA-IR and ApoB levels remained stable or showed a slight increase after the intervention for type 1 responders.

**Table 1:** Changes in anthropometric and metabolic characteristics of type 1 and type 2 responders between week 0 and week 8.

Variable	Type 1 responders		Type 2 responders		*p*-Values		
	Week 0	Week 8	Week 0	Week 8	Group	Visit	Interaction
Weight (kg)	92.6 ± 4.3	93.1 ± 4.3	88.1 ± 7.1	87.5 ± 7.1	0.56	0.51	0.20
BMI (kg/m2)	31.2 ± 1.4	31.4 ± 1.4	29.2 ± 2.4	29.0 ± 2.4	0.45	0.42	0.19
Waist circumference (cm)	102.6 ± 3.3	103.7 ± 3.3	98.7 ± 5.5	96.2 ± 5.5	0.40	0.50	**0.02**
Hip circumference (cm)	113.0 ± 2.8	113.0 ± 2.8	106.8 ± 4.8	106.7 ± 4.8	0.28	0.93	0.88
Waist-Hip ratio	0.91 ± 0.02	0.92 ± 0.02	0.92 ± 0.03	0.90 ± 0.03	0.94	0.52	**0.01**
Systolic blood pressure	116.4 ± 2.4	114.4 ± 2.4	111.2 ± 4.0	110.1 ± 4.0	0.31	0.06	0.71
Diastolic blood pressure	74.9 ± 2.1	73.8 ± 2.2	68.0 ± 3.7	64.6 ± 3.7	0.06	0.14	0.41
ApoB (g/L)	0.93 ± 0.06	0.94 ± 0.06	1.06 ± 0.11	0.9 ± 0.11	0.72	0.20	**0.003**
Total-Cholesterol (mmol/L)	4.54 ± 0.24	4.54 ± 0.24	5.11 ± 0.41	4.78 ± 0.41	0.40	0.54	0.19
TG (mmol/L)	1.61 ± 0.18	1.46 ± 0.19	1.44 ± 0.32	1.19 ± 0.32	0.52	0.24	0.78
HDL-C (mmol/L)	1.18 ± 0.08	1.18 ± 0.08	1.3 ± 0.13	1.27 ± 0.13	0.49	0.80	0.85
LDL-C (mmol/L)	2.61 ± 0.22	2.7 ± 0.22	3.15 ± 0.38	2.96 ± 0.38	0.37	0.79	0.20
Cholesterol HDL-C	4.39 ± 0.33	4.28 ± 0.33	4.06 ± 0.56	3.86 ± 0.56	0.56	0.40	0.79
HbA1C	5.10 ± 0.10	5.10 ± 0.10	5.00 ± 0.10	5.10 ± 0.10	0.66	0.25	0.79
CRP (mg/L)	2.73 ± 0.86	4.2 ± 0.87	2.92 ± 1.46	1.83 ± 1.46	0.53	**0.05**	**0.02**
Fasting glucose (mmol/L)	4.86 ± 0.13	4.78 ± 0.13	4.76 ± 0.21	4.81 ± 0.21	0.88	0.32	0.30
Fasting Insulin (pmol/L)	97.1 ± 10.5	97.4 ± 10.5	87.2 ± 17.6	67.7 ± 17.6	0.35	0.22	**0.02**
Matsuda	4.84 ± 0.56	4.15 ± 0.56	3.59 ± 0.73	5.18 ± 0.73	0.91	0.63	**0.003**
HOMA-IR	2.97 ± 0.58	3.06 ± 0.58	2.96 ± 0.76	2.36 ± 0.76	0.72	0.26	**0.03**
QUICKI	0.33 ± 0.01	0.33 ± 0.01	0.33 ± 0.01	0.34 ± 0.01	0.49	0.41	**0.02**

Data are means ± standard deviation. Type 1 responders (*n* = 17) and type 2 responders (*n* = 5). A linear mixed model adjusted for age, sex, and baseline values was used. BMI, body mass index; ApoB, Apolipoprotein B; TG, triglycerides; HDL-C, high-density lipoprotein cholesterol; LDL-C, low-density lipoprotein cholesterol; HbA1C, glycated hemoglobin; CRP, C-reactive protein.

### 3.4. Most discriminant metabolites between response subgroups

**Figure 3:**
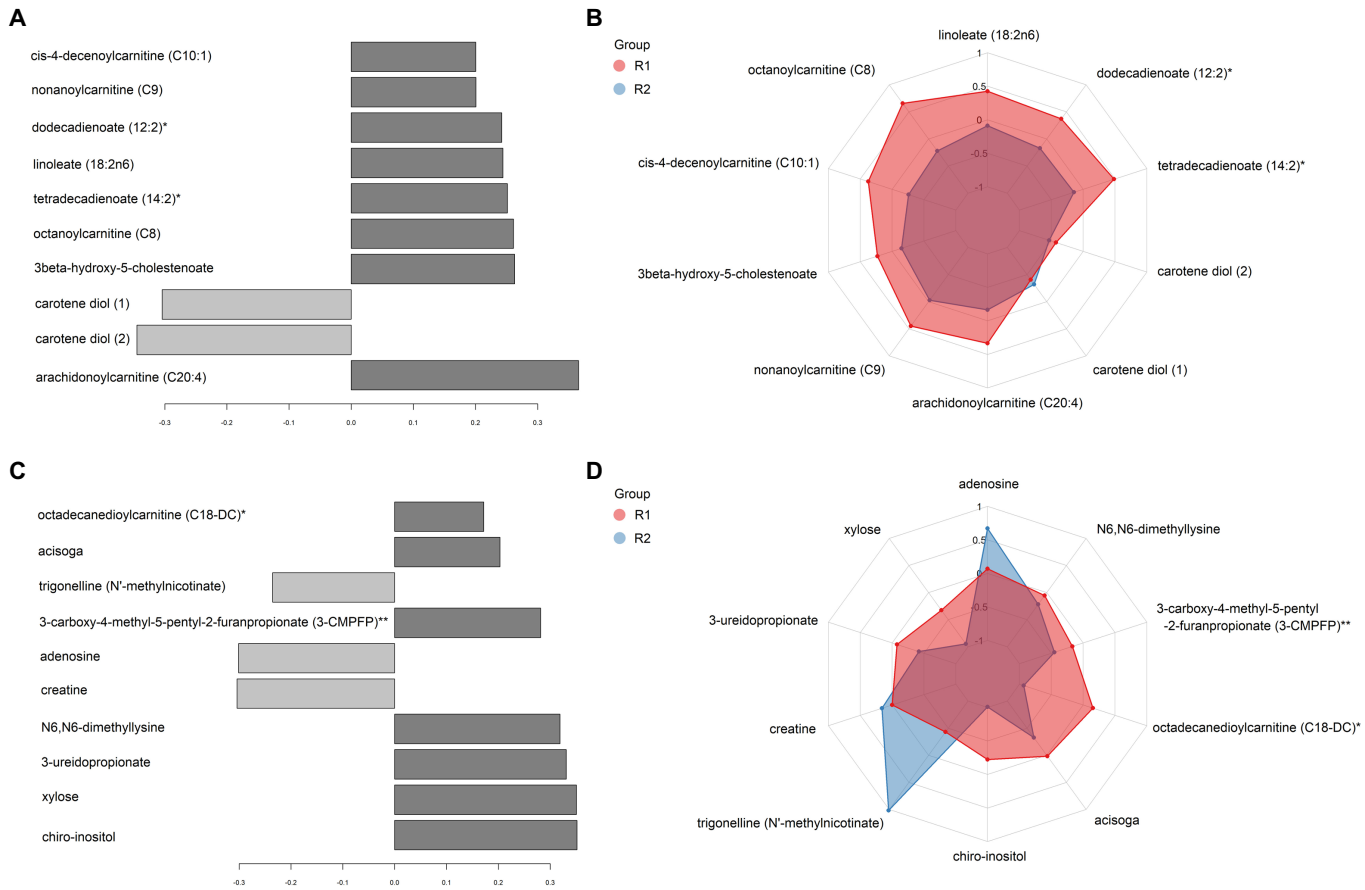
Most discriminant metabolites between type 1 and type 2 responder groups. Panels **(A)** and **(C)** show the ten most discriminant metabolites of component 1 and component 2 of the sparse partial least squares discriminant analysis (sPLS-DA) respectively, ordered from bottom to top by highest loading weight in the discrimination in their respective component. Negative loading weights are shown in light gray and positive loading weights are shown in dark gray. Panels **(B)** and **(D)** show the magnitude of change in metabolite levels for the top ten metabolites in component 1 and component 2 for each response subgroup. Changes for type 1 responders are shown in red and changes for type 2 responders are shown in blue.

We sorted discriminant metabolites obtained through the sPLS-DA based on their loading weight, component by component. The top 10 metabolites of each component are shown in Figure 3. Type 1 responders showed higher average changes on component 1 metabolites than non-responders (Figure 3B). The top 10 metabolites of component 2 are shown in Figure 3C and Figure 3D. Briefly, type 1 responders timepoints were differentiated by metabolites of component 1, and type 2 responders timepoints were differentiated by metabolites by component 2.

**Figure 4:**
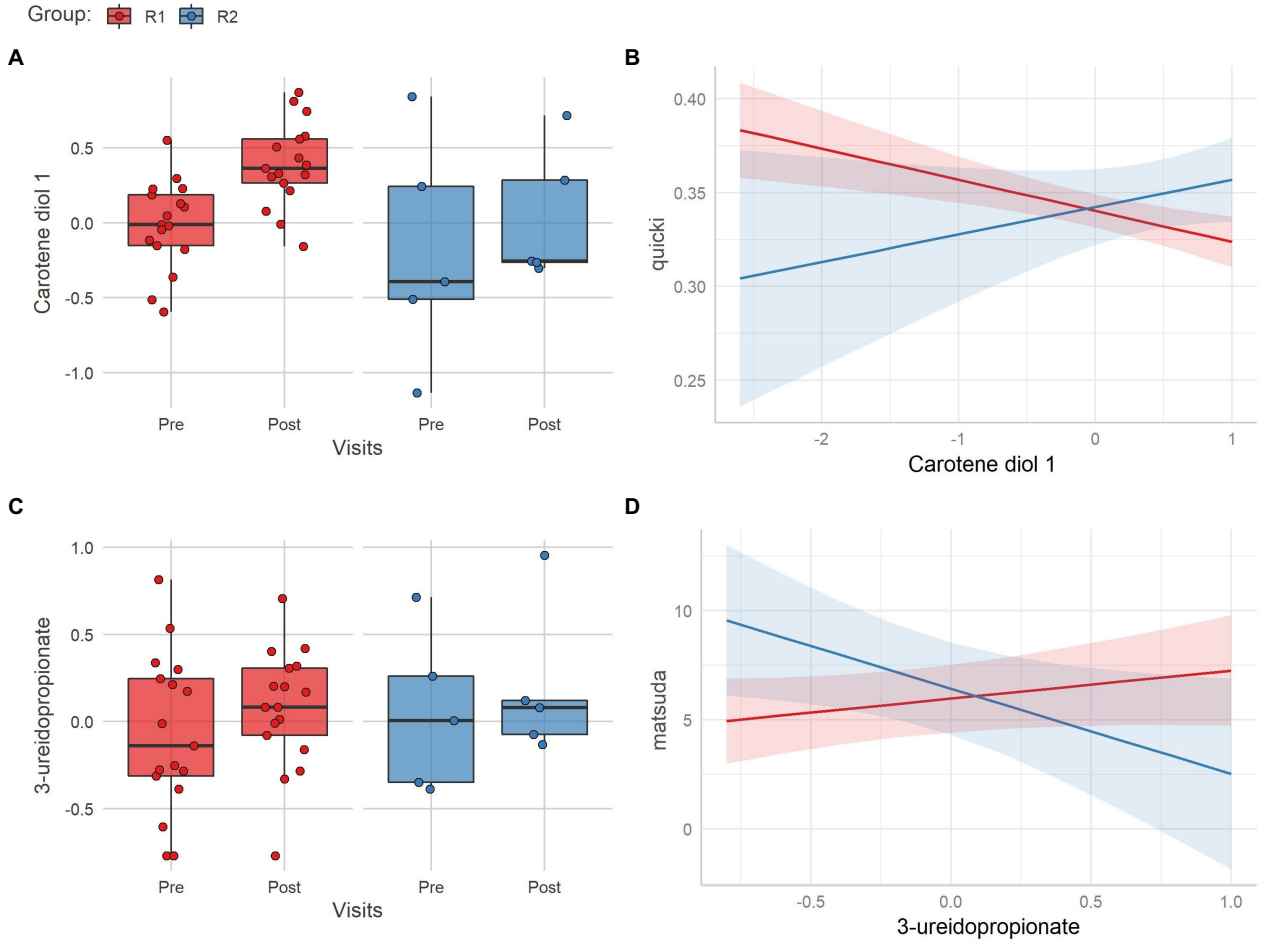
Association between changes in plasma metabolites and metabolic parameters during the intervention by response subgroup. Panels **(A)** and **(C)** show levels of carotene diol 1- and 3-ureidopropionate, respectively, for each response group, before and after the intervention. Panel **(B)** shows the changes of carotene diol 1 in relation to the changes in the QUICKI index for type 1 and type 2 responder groups. Panel **(D)** shows the changes in 3-ureidopropionate in relation to the changes in the Matsuda index for type 1 and type 2 responder groups.

Significant associations were observed between changes in clinical parameters and changes in plasma metabolite levels from both components (Figure 4). The change in fasting insulin and QUICKI index according to the change in carotene diol 1 was significantly different between type 1 and type 2 responders (*p* = 0.04 and *p* = 0.02, respectively). Concretely, we observed a positive association between the increase in carotene diol 1 and fasting insulin levels, and a negative association with the QUICKI index in type 1 responders, while the opposite was observed for type 2 responders, i.e., a decrease in fasting insulin and an increase in the QUICKI index were associated with an increase in carotene diol 1 levels (Figures 4A,B). On the other hand, we found that the change in the Matsuda index according to the change in 3-ureidopropionate was significantly different between type 1 and type 2 responders (*p* = 0.05). A negative association between the increase in the metabolite and the Matsuda index was seen for type 2 responders whereas a positive correlation was observed for type 1 responders (Figures 4C,D).

## 4. Discussion

The most significant finding of this study is that, using a machine learning approach, changes in the levels of plasma metabolites may be used to assess the metabolic responsiveness to raspberry consumption. Concretely, following the classification of study participants into two distinct response subgroups based on the levels of plasma metabolites measured before and after the 8-week raspberry consumption, it is interesting to note significant differences in metabolic health features between the two distinct response subgroups, supporting our clustering approach. In this regard, while positive metabolic responses to the raspberry consumption are already well known (21–24), the results of the present study suggest that a clustering approach of plasma metabolomic data may contribute to explain the interindividual variability observed in metabolic responsiveness to red raspberry consumption.

The use of a metabolomics-based approach for clustering was also particularly useful to assess the adherence to the nutritional intervention. The consumption of xenobiotics present in raspberries led to an increase in such metabolites in the plasma of study participants (25, 26), which were used as raspberry intake markers to identify participants who had a low adherence to the study protocol. The classification algorithm was then trained to predict if a given sample belonged to pre-or post-intervention timepoints and served the purpose of justifying the exclusion of participants suspected of low adherence. This metabolomics-based approach has been used to monitor dietary intake and adherence to a specific diet in recent studies, suggesting that metabolites could be effective biomarkers of food intake (27–31). The second part of the clustering approach was designed to address previous attempts to classify the metabolic responsiveness to an 8-week raspberry consumption, such as a transcriptomics-based approach (12). The metabolomics-based clustering through PLS-DA and sPLS-DA appeared to be relevant, as we observed a homogeneous response within subgroups of participants, as well as a heterogeneous response between subgroups.

Of all the metabolites analyzed in this study, those belonging to the carotenoid family had the most significant influence on metabolic parameters. A regular intake of raspberries has been reported to have positive effects on many metabolic parameters, including improved glucose, insulin, and lipid metabolism as well as reduced inflammation and oxidation (9, 32, 33). These berries contain many carotenoids (34), which have been linked to positive effects on metabolic health. In the present study, two carotenoids, identified as carotene diol 1 and 2, were among the most discriminant metabolites in the clustering analysis, with carotene diol 1 also being significantly linked to opposite changes in fasting insulin and QUICKI index between type 1 and type 2 responders. Circulating plasma carotenoids have been associated with lower inflammation (35–37), including reduced CRP, which we observed in type 2 responders. However, the effects of carotenoids on insulin resistance and the prevention of type 2 diabetes are dichotomic, with studies showing either an inverse association or no association (37). Some studies have reported positive health outcomes on fasting plasma glucose levels and insulin resistance (38) for beta-carotene and lycopene, respectively, whereas other studies have found no correlation between lutein or lycopene and the prevention of type 2 diabetes, but association of alpha-and beta-carotene with type 2 diabetes risk reduction (39). Similarly, carotenoids have also been associated with beneficial lipid and inflammatory responses (40). Many environmental, dietary, physiologic, structural and genetic factors may influence absorption and bioavailability of carotenoids, ranging from gender to hormonal status, interactions with other nutrients or molecules and smoking status, and can affect an individual’s response (41–43). Moreover, previous studies of our team suggest that the heterogeneous association observed between plasma carotenoid concentrations and lipid profiles might be mediated by genetic factors impacting on gene expression and methylation levels (40, 44, 45), which eventually may influence glucose homeostasis differently.

One of the most discriminant metabolites of component 2 in the sPLS-DA was the 3-ureidopropionate, an intermediate in the metabolism of uracil and member of the class of compounds known as ureas (46), and significantly linked to the difference in the Matsuda index between response subgroups. Another propionic derivative which was in the top 10 most discriminant metabolites of component 2 was the 3-carboxy-4-methyl-5-pentyl-2-furanpropionate (3-CMPFP). Propionate is a product of colonic fermentation of dietary fibers (47), which inhibits glucose-induced insulin secretion and glucose decarboxylation in rat pancreatic cells (48). More recent studies have confirmed that propionate improves beta-cell function in humans (49) and improved insulin sensitivity (50), which can be linked to the well documented health effects of dietary fibers on glucose homeostasis (51, 52). Discrepancies reported in the literature around the impact of raspberry consumption on metabolic health are reflected in the present study. Accordingly, most of the significant effects observed in type 2 responders were generally beneficial on metabolic health. However, the increase in 3-ureidopropionate during raspberry consumption had opposing effects. Concretely, increasing levels of 3-ureidopropionate were associated with an increase in the Matsuda index in type 1 responders, and therefore with an improvement in insulin sensitivity. In contrast, type 2 responders sustained a decrease in Matsuda index values with increasing 3-ureidopropionate levels, and a deterioration of their insulin sensitivity. However, overall insulin sensitivity of type 2 responders after the intervention was improved. Although these results are preliminary due to the small numbers on which they are based, they reflect the interindividual variability observed in metabolic responsiveness to a nutritional intervention, possibly attributable to divergent capacity to metabolize, absorb or use these metabolites. On the other hand, chiro-inositol was the most discriminant metabolite of component 2. It has been reported to serve a purpose in the mediation of insulin action, and low concentrations have been linked to increased insulin resistance (53, 54). Xylose, the second most discriminant metabolite of component 2, has been linked to improved blood glucose levels regulation by selectively inhibiting the activity of sucrase (55, 56), and may have been a factor in the improved insulin sensitivity of type 2 responders. The different responses we observed between type 1 and type 2 responders may have different causes. Studies focusing on fruits and vegetables consumption found that many factors could influence the heterogeneity of individuals’ response: health status, excess weight, chronic inflammation and hypertension can affect the absorption and metabolism of biomarkers. This may explain the varying effectiveness of the intervention in the at risk of metabolic syndrome population of this study (57). Other studies have explored the different responses in individuals to the same meal, finding many determinants of postprandial metabolism such as glycemic response and triglyceride and insulin concentrations (58). Concentrations of specific enzymes and some polymorphisms and mutations affecting key genes may also influence individual metabolic response to many nutrients and therefore the presence of their metabolites in plasma samples (59). Metabolomics alone cannot fully explain the different responses we observed between the two subgroups, and future studies may use a multi-omics approach to further understand interindividual variability and its causes.

The small sample size of the present study as well as the low number of type 2 responders can be considered as a limitation and results should be interpreted with caution. The generalization of clustering results therefore requires further studies in larger, independent study samples to confirm the present findings. The subgroup of type 2 responders with positive health outcomes consisted of only five people, which limits the impact of these results. Another limitation was the absence of polyphenols and polyphenol-derived metabolites in the Metabolon database of metabolites used in this study. The inclusion of these molecules could have revealed more meaningful interactions between metabolites and changes in clinical variables, allowing us to understand the distinct metabolic effects of red raspberry consumption. Moreover, the absence of a control group was also a limitation. The participants of the study were required to limit their berry consumption and maintain consistent health habits during a 2-week run-in period, leading to raspberry supplementation as the primary dietary change during the intervention. However, since we do not have data from the control group, these observations should be considered exploratory in nature and further studies utilizing randomized designs will be necessary to validate our findings. Despite the small sample size, some significant interactions between metabolites and clinical features were still found, opening the door to more in-depth studies on specific metabolites. Moreover, the use of metabolomics are herein revealed as particularly promising in assessing dietary intake biomarkers in conjunction with self-reported dietary assessment methods such as food frequency questionnaires, which alone are prone to a certain error (60, 61). Such results warrant further investigation into large study samples to confirm the potential of xenobiotics as a marker of adherence to nutritional interventions.

In conclusion, this metabolomics-based clustering approach derived from an 8-week raspberry consumption allowed to develop a framework to address the impact of the interindividual variability on the metabolic responsiveness to raspberry consumption. This approach paves the way to future studies focused on further understanding the role of plasma metabolites in identifying individuals more prone to take advantage from a nutritional intervention aimed at having beneficial health effects. This framework may then be extrapolated to understand other diseases and conditions, and further enhance the development of precision nutrition.

## Data availability statement

The raw data supporting the conclusions of this article will be made available by the authors, without undue reservation.

## Ethics statement

The studies involving human participants were reviewed and approved by Université Laval Ethics Committee (CER-Université Laval 2017-218). The patients/participants provided their written informed consent to participate in this study.

## Author contributions

VB prepared the manuscript. JT-M, VB, and RS-C conducted the metabolomic and statistical analyses. MV designed and supervised the study. VG coordinated the study and PC assumed the medical supervision. MV, JT-M, RS-C, VG, GP, PC, DR, CC, and AM reviewed/revised and approved the final manuscript. All authors contributed to the article and approved the submitted version.

## Funding

The *Washington Red Raspberry Commission* (WRRC) funded the present study. WRRC was not involved in the study design, data collection, interpretation of results, decision to publish, or redaction of the manuscript.

## Conflict of interest

The authors declare that the research was conducted in the absence of any commercial or financial relationships that could be interpretated as a potential conflict of interest.

## Publisher’s note

All claims expressed in this article are solely those of the authors and do not necessarily represent those of their affiliated organizations, or those of the publisher, the editors and the reviewers. Any product that may be evaluated in this article, or claim that may be made by its manufacturer, is not guaranteed or endorsed by the publisher.
