# Supplementary material for: A discriminant analysis of plasma metabolomics for the assessment of metabolic responsiveness to red raspberry consumption
Source: Front Nutr. 2023 Mar 23;10:1104685. doi: 10.3389/fnut.2023.1104685 (PMC10130762; doi:10.3389/fnut.2023.1104685)
Supplement: Supplementary file 1 [file Data_Sheet_1.docx]

**Supplementary Tables**

**Supplementary Table 1. Estimated nutritional composition of consumed raspberries.**

Macro- and micronutrient intake from raspberry consumption were estimated by using a food frequency questionnaire developed in the Institute of Nutrition and Functional Foods (INAF) and validated in a French-Canadian population (1). Evaluation of nutrient intakes derived from the food frequency questionnaire was performed using the Nutrition Data System for Research (NDS-R) software, developed by the Nutrition Coordination Center of the University of Minnesota, and using the Food and Nutrient Database 31. This database includes more than 16000 food items for which the complete nutritional value of 112 nutrients is included (2).

| Nutrient | Quantity (for 280g) |
| --- | --- |
| Energy (kcal) | 158.24 |
| Alcohol (g) | 0 |
| Total Fat (g) | 1.36 |
| Total Saturated Fatty Acids (SFA) (g) | 0.08 |
| Total Monounsaturated Fatty Acids (MUFA) (g) | 0.16 |
| Total Polyunsaturated Fatty Acids (PUFA) (g) | 0.72 |
| Total Trans-Fatty Acids (TRANS) (g) | 0 |
| Total Vitamin A Activity (Retinol Equivalents) (mcg) | 24.56 |
| Beta-Carotene (provitamin A carotenoid) (mcg) | 130.88 |
| Alpha-Carotene (provitamin A carotenoid) (mcg) | 26.96 |
| Beta-Cryptoxanthin (provitamin A carotenoid) (mcg) | 6.56 |
| Lutein + Zeaxanthin (mcg) | 707.88 |
| Lycopene (mcg) | 0.00 |
| Total Vitamin A Activity (International Units) (IU) | 246.00 |
| Retinol (mcg) | 0 |
| Vitamin D (calciferol)(mcg) | 0 |
| Total Vitamin E Activity (total alpha-tocopherol equivalents) (mg) | 1.72 |
| Alpha-Tocopherol (mg) | 1.40 |
| Beta-Tocopherol (mg) | 0.04 |
| Gamma-Tocopherol (mg) | 2.76 |
| Delta-Tocopherol (mg) | 3.56 |
| Vitamin K (phylloquinone)(mcg) | 8.64 |
| Vitamin C (ascorbic acid) (mg) | 92.28 |
| Thiamin (vitamin B1) (mg) | 0.12 |
| Riboflavin (vitamin B2) (mg) | 0.20 |
| Niacin (vitamin B3) (mg) | 1.48 |
| Pantothenic acid (mg) | 0.76 |
| Vitamin B-6 (pyridoxine, pyridoxyl, & pyridoxamine) (mg) | 0.20 |
| Vitamin B-12 (cobalamin)(mcg) | 0 |
| Dietary Folate Equivalents (mcg) | 61.12 |
| Calcium (mg) | 55.64 |
| Magnesium (mg) | 40.36 |
| Iron (mg) | 1.32 |
| Sodium (mg) | 5.92 |
| Potassium (mg) | 469.12 |
| Total Protein (g) | 2.32 |
| Total Carbohydrate (g) | 38.76 |
| Fructose (g) | 10.20 |
| Galactose (g) | 0 |
| Glucose (g) | 10.76 |
| Lactose (g) | 0 |
| Maltose (g) | 0.40 |
| Sucrose (g) | 3.60 |
| Starch (g) | 0.00 |
| Total Dietary Fiber (g) | 14.28 |
| Soluble Dietary Fiber (g) | 2.32 |
| Insoluble Dietary Fiber (g) | 11.96 |
| Water (g) | 294.08 |

**Supplementary Table 2. Anthropometric and metabolic characteristics of type 1 and type 2 responders at week 0.**

Data are means ± standard deviation. Type 1 responders (n=17) and type 2 responders (n=5). Sex is presented as counts. Abbreviations: BMI, body mass index; ApoB, Apolipoprotein B; TG, triglycerides; HDL-C, high-density lipoprotein cholesterol; LDL-C, low-density lipoprotein cholesterol; HbA1C, glycated hemoglobin; CRP, C-reactive protein.

| Variable |  | Type 1 responders | | | Type 2 responders | | | *P-value* |
| --- | --- | --- | --- | --- | --- | --- | --- | --- |
| Sex (women/men) |  | 13/4 |  |  | 2/3 |  |  | 0.14 |
| Age (years) |  | 31.9 | ± | 9.1 | 34.0 | ± | 14.9 | 0.70 |
| Weight (kg) |  | 89.5 | ± | 15.2 | 86.4 | ± | 17.2 | 0.72 |
| BMI (kg/m2) |  | 30.1 | ± | 5.5 | 29.3 | ± | 2.1 | 0.74 |
| Waist circumference (cm) |  | 96.3 | ± | 13.6 | 100.6 | ± | 12.0 | 0.54 |
| Hip circumference (cm) |  | 112.0 | ± | 10.0 | 106.5 | ± | 6.4 | 0.26 |
| Waist-Hip ratio |  | 0.86 | ± | 0.11 | 0.94 | ± | 0.09 | 0.13 |
| Systolic blood pressure |  | 113.3 | ± | 10.2 | 111.5 | ± | 12.8 | 0.74 |
| Diastolic blood pressure |  | 73.0 | ± | 8.1 | 68.1 | ± | 11.8 | 0.30 |
| ApoB (g/L) |  | 0.87 | ± | 0.24 | 1.08 | ± | 0.24 | 0.11 |
| Total-Cholesterol (mmol/L) |  | 4.52 | ± | 0.93 | 5.11 | ± | 0.92 | 0.22 |
| TG (mmol/L) |  | 1.30 | ± | 0.65 | 1.50 | ± | 0.59 | 0.53 |
| HDL-C (mmol/L) |  | 1.39 | ± | 0.42 | 1.24 | ± | 0.27 | 0.47 |
| LDL-C (mmol/L) |  | 2.53 | ± | 0.82 | 3.18 | ± | 0.98 | 0.15 |
| Cholesterol HDL-C |  | 3.64 | ± | 1.69 | 4.25 | ± | 1.25 | 0.46 |
| HbA1C |  | 5.00 | ± | 0 | 5.00 | ± | 0 | 0.94 |
| CRP (mg/L) |  | 3.23 | ± | 3.10 | 2.80 | ± | 3.22 | 0.79 |
| Fasting glucose (mmol/L) |  | 4.73 | ± | 0.46 | 4.81 | ± | 0.59 | 0.75 |
| Fasting Insulin (pmol/L) |  | 75.6 | ± | 46.6 | 92.2 | ± | 27.0 | 0.46 |
| Matsuda |  | 5.11 | ± | 2.54 | 3.56 | ± | 1.35 | 0.29 |
| HOMA-IR |  | 2.71 | ± | 2.05 | 3.08 | ± | 1.22 | 0.75 |
| QUICKI |  | 0.34 | ± | 0.02 | 0.32 | ± | 0.02 | 0.19 |

**Supplementary Figures**

**Supplementary Figure 1**


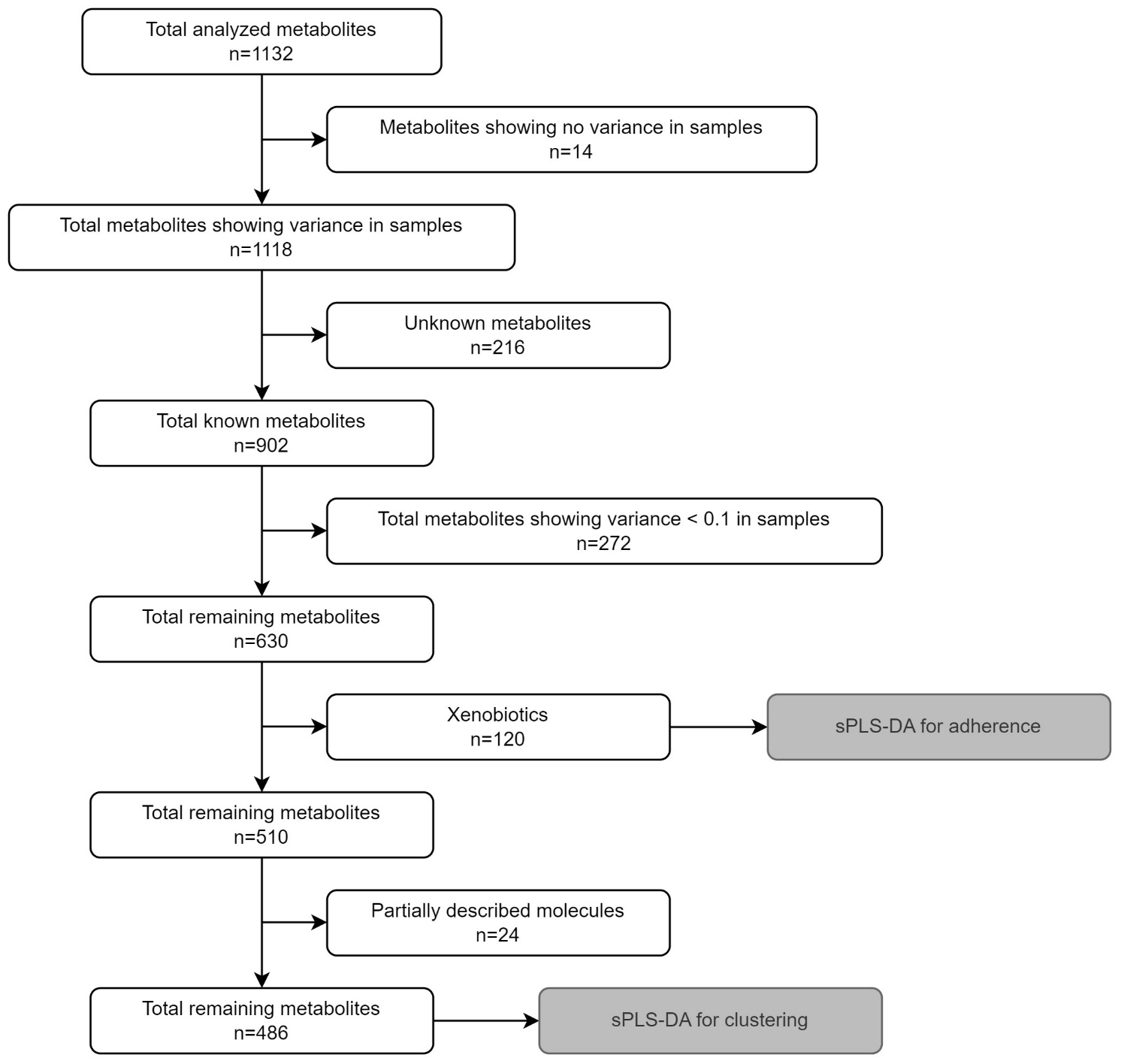


**Supplementary Figure 1. Metabolite filtering for adherence and clustering analyses.**

**Supplementary Figure 2**


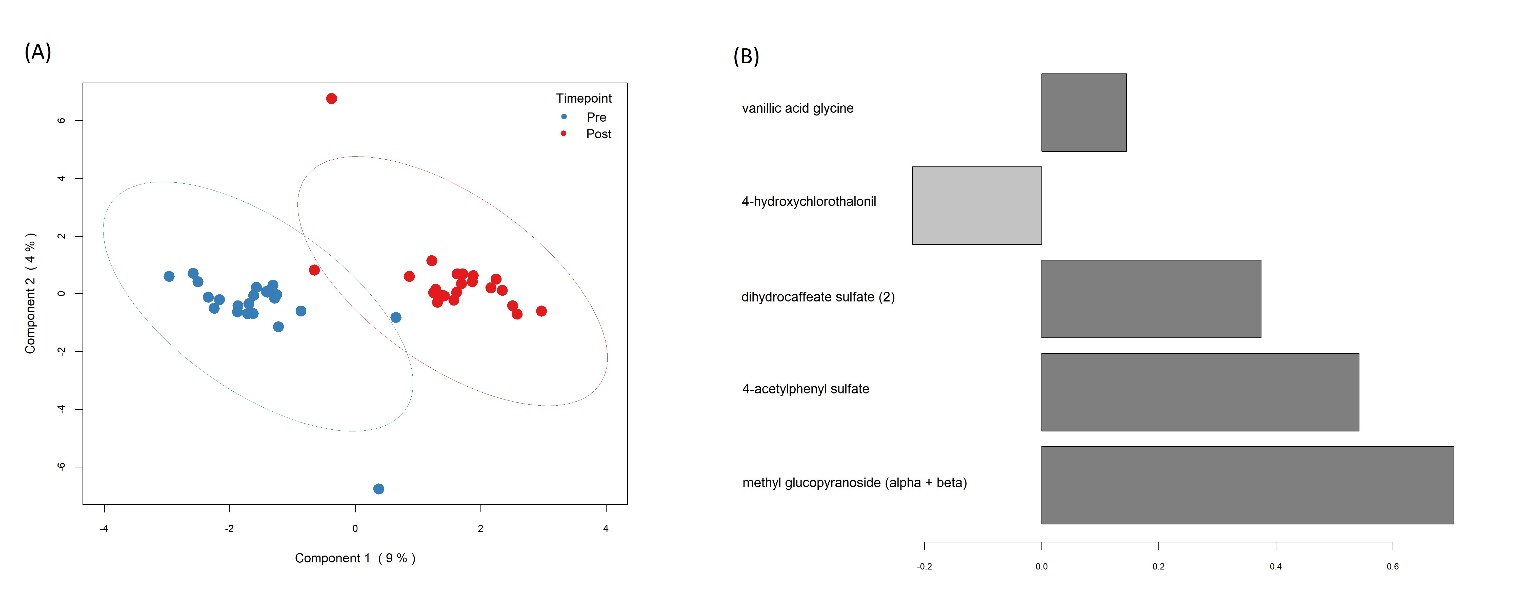


**Supplementary Figure 2. Adherence to the nutritional intervention and outlier detection.**

Panel **(A)** shows the partial least squares discriminant analysis (sPLS-DA) use to confirm adherence to the nutritional intervention by discriminating trial visits, before and after raspberry consumption, using only xenobiotics (n=120). The two identified outliers are represented by the two most extreme dots, and the two most central dots. Panel **(B)** shows the top five most discriminant metabolites for component 1, ordered from bottom to top by highest loading weight in the discrimination. Negative loading weights are shown in light gray and positive loading weights are shown in dark gray.

**Supplementary Figure 3**

**
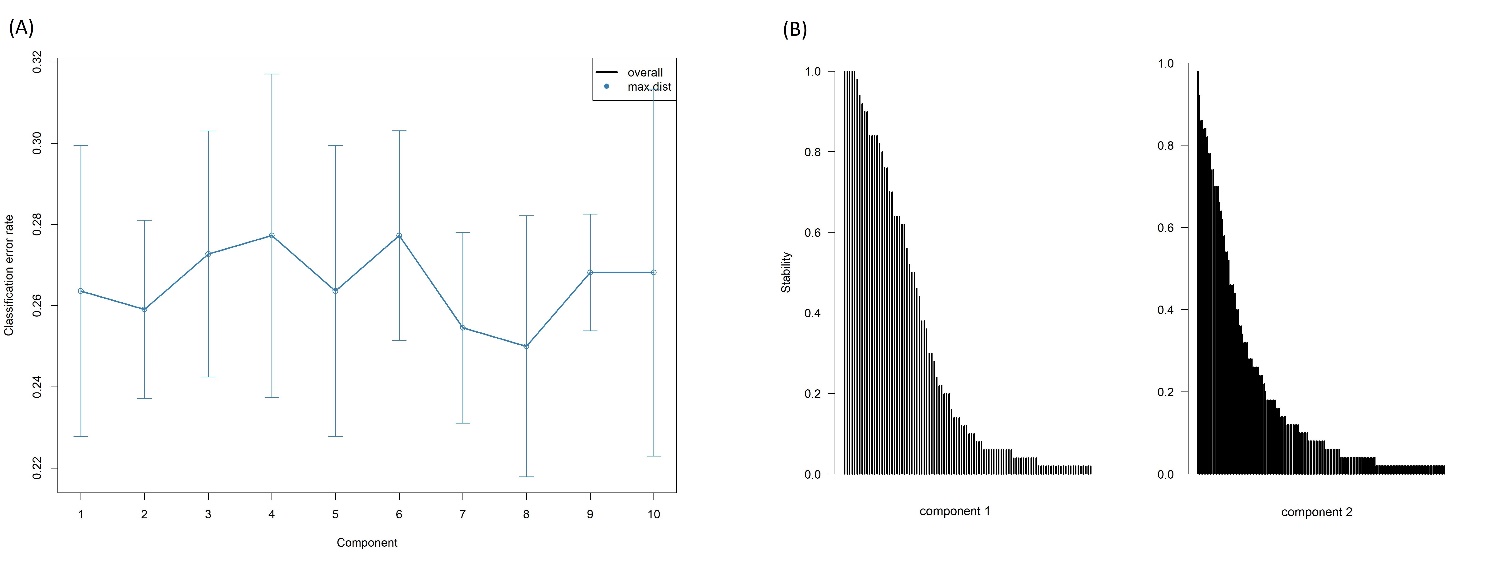
**

**Supplementary Figure 3. Performance evaluation of the sPLS-DA model**

Panel **(A)** shows the Performance evaluation of the sparse partial least squares discriminant analysis (sPLS-DA) model used to show type 1 and type 2 responder groups. Panel **(B)** shows the stability of the selected metabolites in the sPLS-DA model.

**References**

1. Schakel SF, Sievert YA, Buzzard IM. Sources of data for developing and maintaining a nutrient database. J Am Diet Assoc. 1988 Oct;88(10):1268–71.

2. Goulet J, Nadeau G, Lapointe A, Lamarche B, Lemieux S. Validity and reproducibility of an interviewer-administered food frequency questionnaire for healthy French-Canadian men and women. Nutr J. 2004 Sep 13;3:13.
