# Supplementary figures and images for: A discriminant analysis of plasma metabolomics for the assessment of metabolic responsiveness to red raspberry consumption
Source: Front Nutr. 2023 Mar 23;10:1104685. doi: 10.3389/fnut.2023.1104685 (PMC10130762; doi:10.3389/fnut.2023.1104685)

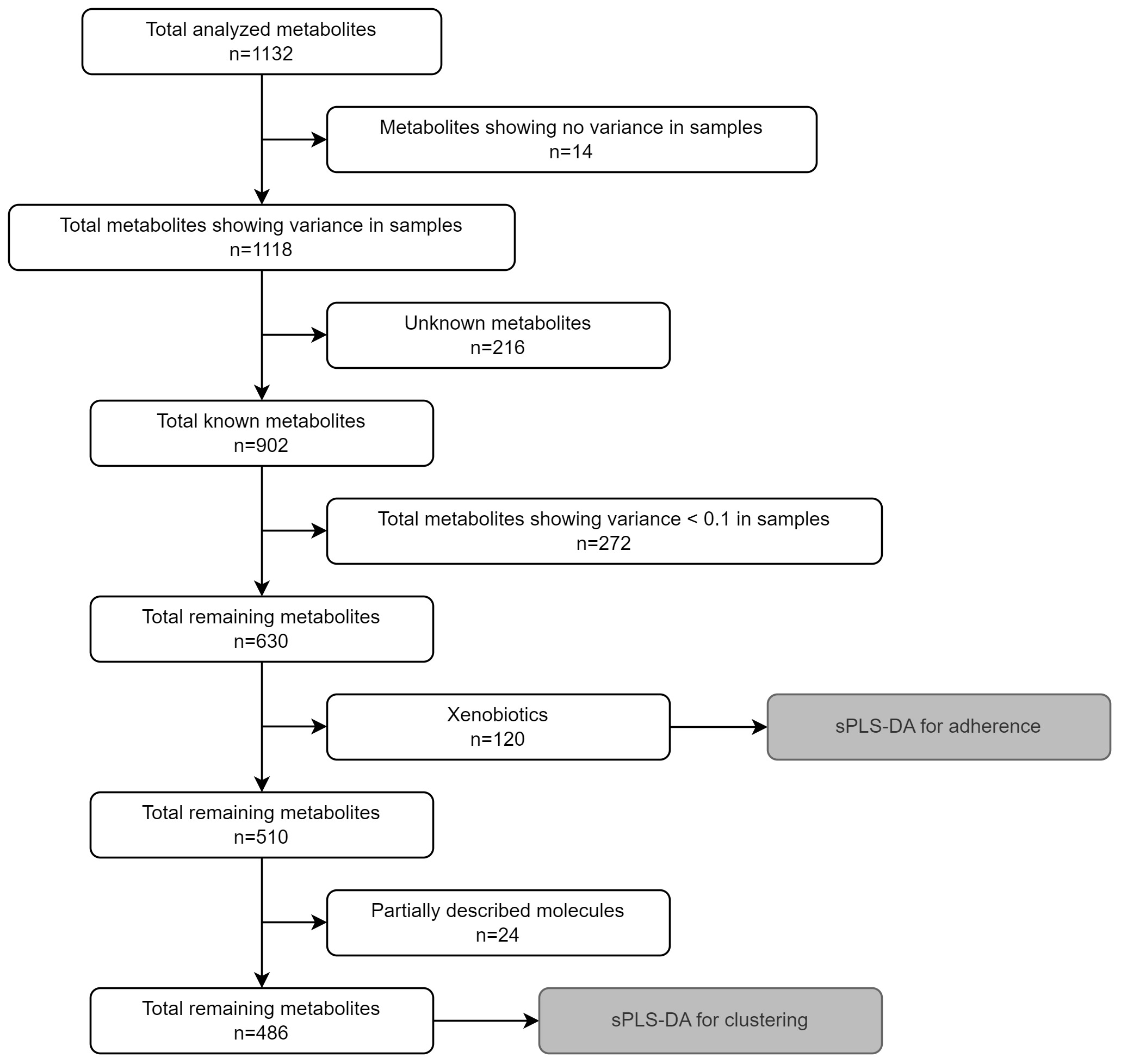

Supplement: Supplementary file 2 [file Image_1.JPEG]

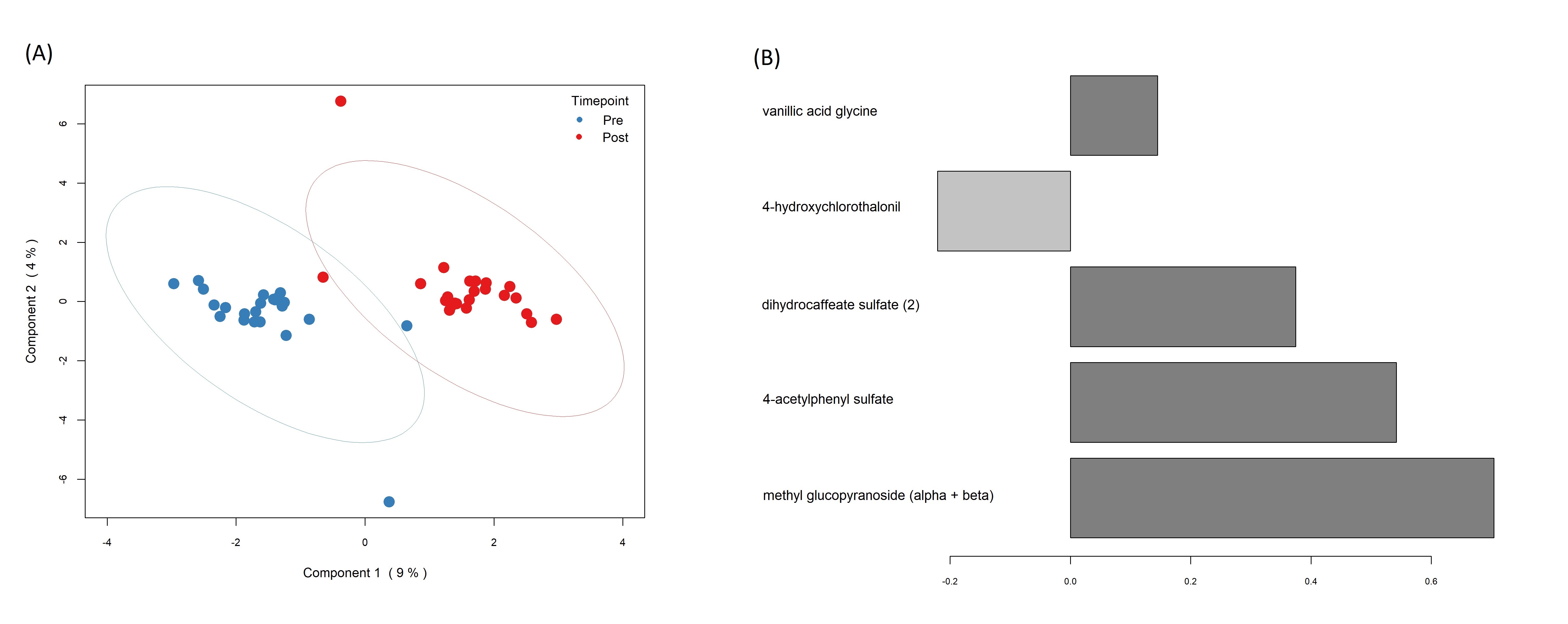

Supplement: Supplementary file 3 [file Image_2.JPEG]

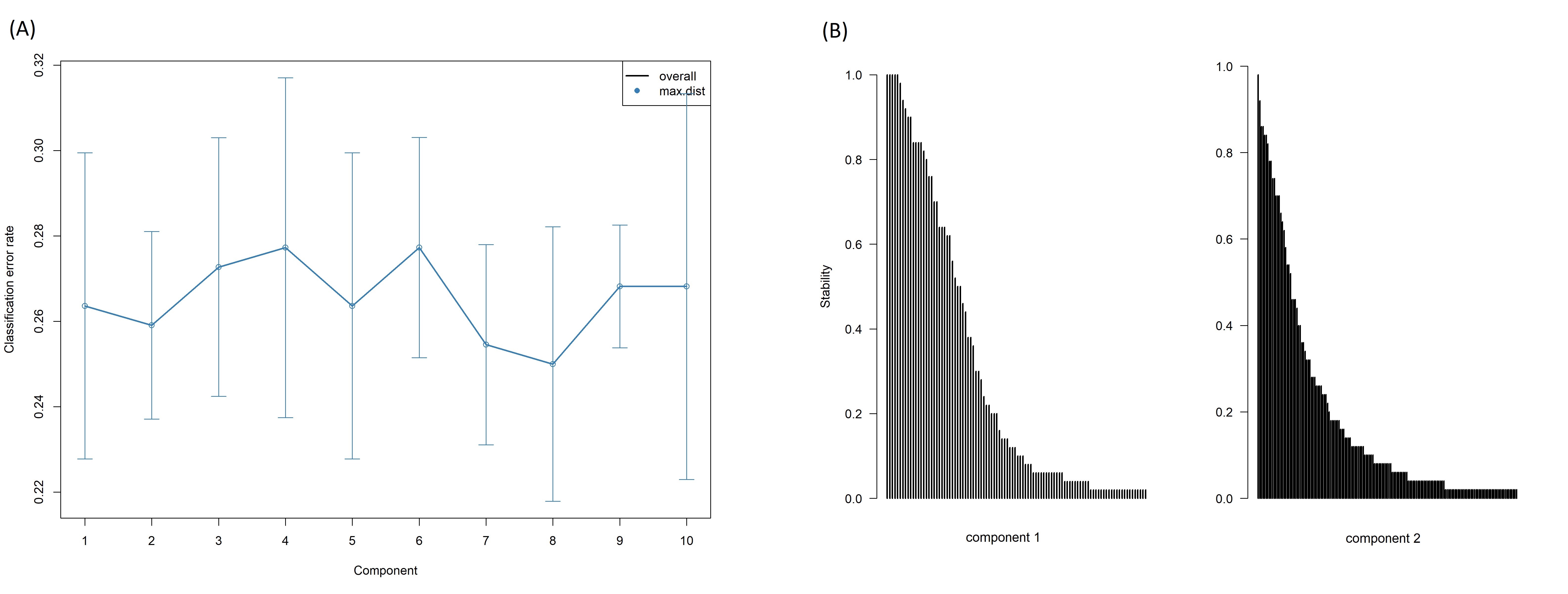

Supplement: Supplementary file 4 [file Image_3.JPEG]
